# Expression and Function of Interleukin-1β-Induced Neutrophil Gelatinase-Associated Lipocalin in Renal Tubular Cells

**DOI:** 10.1371/journal.pone.0166707

**Published:** 2016-11-16

**Authors:** Tadayoshi Konno, Rei Nakano, Ryo Mamiya, Hisashi Tsuchiya, Taku Kitanaka, Shinichi Namba, Nanako Kitanaka, Ken Okabayashi, Takanori Narita, Hiroshi Sugiya

**Affiliations:** Laboratory of Veterinary Biochemistry, Department of Veterinary Medicine, Nihon University College of Bioresource Sciences, 1866 Kameino, Fujisawa, Kanagawa, 252–0880, Japan; Center for Molecular Biotechnology, ITALY

## Abstract

Acute kidney injury (AKI) is characterized by a sudden loss of renal function. Early recognition of AKI, especially in critically ill patients, is essential for adequate therapy. Currently, neutrophil gelatinase-associated lipocalin (NGAL) is considered to be an effective biomarker of AKI; however, the regulation of its expression and function in renal tubular cells remains unclear. In this study, we investigated the regulation of the expression and function of NGAL in IL-1β-treated Madin–Darby canine kidney (MDCK) cells as a model of renal tubular cells. IL-1β induced a disturbance in the localization of E-cadherin and zonaoccludin-1 (ZO-1). The transepithelial electrical resistance (TER) also decreased 5 days after IL-1β treatment. IL-1β induced NGAL mRNA expression and protein secretion in a time- and dose-dependent manner, which occurred faster than the decrease in TER. In the presence of ERK1/2 and p38 inhibitors, IL-1β-induced NGAL mRNA expression and protein secretion were significantly attenuated. In the presence of recombinant NGAL, IL-1β-induced disturbance in the localization of E-cadherin and ZO-1 was attenuated, and the decrease in TER was partially maintained. These results suggest that NGAL can be used as a biomarker for AKI and that it functions as a protector from AKI.

## Introduction

Acute kidney injury (AKI) is defined as a rapid decrease in the glomerular filtration rate and leads to high patient mortality. Structural damage and death of renal tubular cells are observed in AKI, and the damaged cells may release inflammatory mediators. During the development and progression of AKI, renal tubular cell death and inflammation may influence the severity and prognosis of AKI [1–4].

Inflammatory mediators are synthesized by renal tubular cells and induce tubular dysfunction in a paracrine and autocrine manner [5]. Interleukin-1 (IL-1) family members such as IL-1β and IL-18 are associated with the development and progression of AKI [6–8]. IL-1β is initially synthesized as the inactive precursor pro-IL-1β, whose maturation is achieved through inflammasomes and caspase-1 [9,10]. Inflammasomes are protein assemblies in the cytoplasm that consist of three main components: a sensor protein (receptor), an adaptor protein, and caspase-1 [11]. They mediate the activation of a highly inflammatory form of cell death, pyroptosis [8,10]. In renal tubular cells, inflammasome-mediated caspase-1 activation and IL-1β generation are induced by several extra- and intracellular stimuli such as ischemic-reperfusion injury, adenosine triphosphate (ATP), hypotonic stress, uric acid crystals, mitochondrial dysfunction, and lysosomal rupture [11–14].

Several biomarkers have been investigated for the early detection of AKI. Serum creatinine and blood urea nitrogen (BUN) have routinely been used as biomarkers of AKI. However, they are insufficient for the early detection of AKI, because serum creatinine and BUN levels rise only after severe histopathological damage in the kidney. Furthermore, the levels of both biomarkers are influenced by non-renal factors, including age, sex, muscle mass, nutritional status, infection, volume of distribution, and medications [15]. Currently, neutrophil gelatinase-associated lipocalin (NGAL) is considered an effective biomarker of AKI [16, 17]. NGAL, a 25-kDa protein belonging to the lipocalin family, has a high affinity for siderophores and is involved in the neutrophilic response to infections through iron chelation or delivering [16–18]. NGAL is thought to be an acute-phase protein, whose expression is upregulated in various types of epithelial cells under diverse inflammatory diseases [19, 20]. However, the regulation of the expression and function of NGAL in renal tubular epithelial cells remains unclear. In this study, we used Madin–Darby canine kidney (MDCK) cells as a model of renal tubular cells and investigated the regulation of the expression and function of NGAL in IL-1β-induced renal tubular cells.

## Materials and Methods

### Materials

Canine recombinant IL-1β and NGAL were purchased from Kingfisher Biotech, Inc. (Saint Paul, MN) and United States Biological (Salem, MA), respectively. TRIzol, anti-zonaoccludin-1 (ZO-1) mouse monoclonal antibody (Clone: ZO-1-1A12), Alexa Fluor 488-conjugated F(ab′)2 fragments of goat anti-rabbit IgG (H+L), Alexa Fluor 594-conjugated F(ab′)2 fragments of goat anti-mouse IgG (H+L), TO-PRO-3-iodide, and ProLong Gold Antifade Reagent were purchased from Life Technologies Co. (Carlsbad, CA). PrimeScript RT Master Mix and SYBR Premix Ex Taq II were obtained from TaKaRa Bio Inc. (Shiga, Japan). Rabbit monoclonal antibodies against E-cadherin (Clone: 24E10) were purchased from Cell Signaling Technology Japan (Tokyo, Japan). The mitogen-activated protein kinase (MAPK) inhibitors FR180204, SB239063, SP600125, and U0126, and the IκB kinase inhibitors BAY-117082 and 2-[(aminocarbonyl)amino]-5-(4-fluorophenyl)-3-thiophenecarboxamide (TPCA-1) were purchased from Sigma-Aldrich Inc. (St Louis, MO). The NGAL assay kit was purchased from BioPorto Diagnostics A/S (Hellerup, Denmark). StatMate IV was purchased from ATMS (Tokyo, Japan). Culture plates, dishes, and flasks were obtained from Thermo Fisher Scientific, Inc. (St. Waltham, MA).

### Cell culture

MDCK (NBL-2) cells were purchased from the Japanese Collection of Research Bioresources Cell Bank (Osaka, Japan). The cells were static-cultured in an incubator at 5% CO_2_ and 37°C using Dulbecco’s modified Eagle’s medium with low glucose (DMEM-LG; Wako Pure Chemical Industries, Ltd., Osaka, Japan) supplemented with 10% fetal bovine serum (FBS). The culture medium was changed twice a week. When the cells reached approximately 90% confluence, they were detached from the culture flask using 0.25% trypsin-EDTA. The collected cells were seeded at a density of 5 × 10^5^ cells/75-cm^2^ culture flask. Tenth-passage MDCK cells were used for all the following experiments.

### IL-1β treatment

MDCK cells were seeded at a density of 5 × 10^4^ cells/2 ml culture medium/well into 6-well culture plates or 5 × 10^5^ cells/12 ml culture medium/90-mm dish and cultured for 72 h. After pre-treatment with DMEM-LG and 2% FBS for 12 h, the cells were treated with IL-1β (concentrations were described in figure legends).

### Real-time RT-PCR

Total RNA was extracted from MDCK cells using the TRIzol reagent. First-strand cDNA synthesis was carried out with 500 ng of total RNA using PrimeScript RT Master Mix. Real-time RT-PCR was performed with 2 μl of the first-strand cDNA in 25 μl (total reaction volume) containing SYBR Premix Ex Taq II and primers specific for canine NGAL or TATA box-binding protein (TBP), a housekeeping protein. Table 1 shows the primer sequences used for real-time RT-PCR. No-template controls and no-reverse transcription controls were included as 2 μl each of RNase/DNA-free water and RNA samples. PCR was conducted using a Thermal Cycler Dice Real Time System II (TaKaRa Bio Inc.). The PCR profile consisted of 1 cycle of denaturation at 95°C for 30 s, 40 cycles of denaturation at 95°C for 5 s and annealing and extension at 60°C for 30 s. The results were analyzed using the second derivative method and the comparative cycle threshold (ΔΔCt) method with software for real-time RT-PCR analysis (TP900 DiceRealTime v4.02B; TaKaRa Bio Inc.). Amplification of TBP from the same amount of cDNA was used as an endogenous control, and the amplification of the cDNA from MDCK cells (time: 0) was used as a calibrator standard.

**Table 1:** Primers used for Real-time RT-PCR.

Gene	Gene bank ID	Primer sequences	Size (bps)	position
*NGAL*	XM_857229.2	F: 5ʹ-CACCTCCACCCTACTCAGGAATG-3ʹ	95	290~384
		F: 5ʹ-GAATGTCGCCCAGGCTGAA-3ʹ		
*TBP*	XM_863452	F: 5'-ACTGTTGGTGGGTCAGCACAAG-3'	124	472~654
		R: 5'-ATGGTGTGTACGGGAGCCAAG-3'		

### Immunocytochemistry

MDCK cells were seeded at a density of 5 × 10^4^ cells/2 ml culture medium/well into a 35-mm glass base dish (Iwaki, Tokyo, Japan) and treated with IL-1β as described above. The cells were fixed in 4% paraformaldehyde (Nacalai Tesque Inc., Kyoto, Japan) for 15 min and processed for immunocytochemistry to examine the cellular localization of F-actin and ZO-1. The fixed cells were permeabilized by incubation with 0.2% Triton X-100 (Sigma-Aldrich Inc.) for 15 min at room temperature. Non-specific antibody reactions were blocked for 30 min with Block Ace (DS Pharma Biomedical, Osaka, Japan). The cells were then incubated for 90 min at room temperature with the following primary antibodies: anti-E-cadherin rabbit antibody [1:500] or anti-ZO-1 mouse antibody [1:500]. After the cells were washed with PBS containing 0.2% polyoxyethylene (20) sorbitan monolaurate, they were incubated and visualized with Alexa Fluor 488-conjugated F(ab′)2 fragments of goat anti-rabbit IgG (H+L) [1:1000], Alexa Fluor 594-conjugated F(ab′)2 fragments of goat anti-mouse IgG (H+L) [1:1000], and TO-PRO-3-iodide [1:1000] for 60 min in the dark at room temperature. The cells were also incubated with only secondary antibodies as a control for nonspecific binding of the antibodies. These samples were washed thrice with PBS containing 0.2% polyoxyethylene (20) sorbitan monolaurate, dried, mounted with ProLong Gold Antifade Reagent, and visualized using a confocal laser scanning microscope (LSM-510; Carl Zeiss AG, Oberkochen, Germany).

### ELISA

MDCK cells were treated with IL-1β as described above, and the culture medium was collected. The concentration of NGAL in the medium was measured using an ELISA kit according to the manufacturer’s instructions.

### Measurement of transepithelial electrical resistance

Cells were plated at a density of 5 × 10^4^ cells/cm^2^ on Transwell filter inserts, and the electrical resistance across the cell monolayer was measured using a Millicell ERS-2 epithelial volt-ohm meter (Millipore, Billerica, MA) according to the manufacturer’s instructions. Transepithelial electrical resistance (TER) values were obtained by subtracting the resistance of the blank filter [21].

### Statistical analysis

The data were calculated as mean ± standard error (SE). Statistical analyses were performed using StatMate IV. The data were analyzed using one-way analysis of variance (ANOVA), except for the time-course study, where two-way ANOVA was used.

## Results

### IL-1β affects the localization of E-cadherin and ZO-1 and their functions in MDCK cells

**Fig 1:**
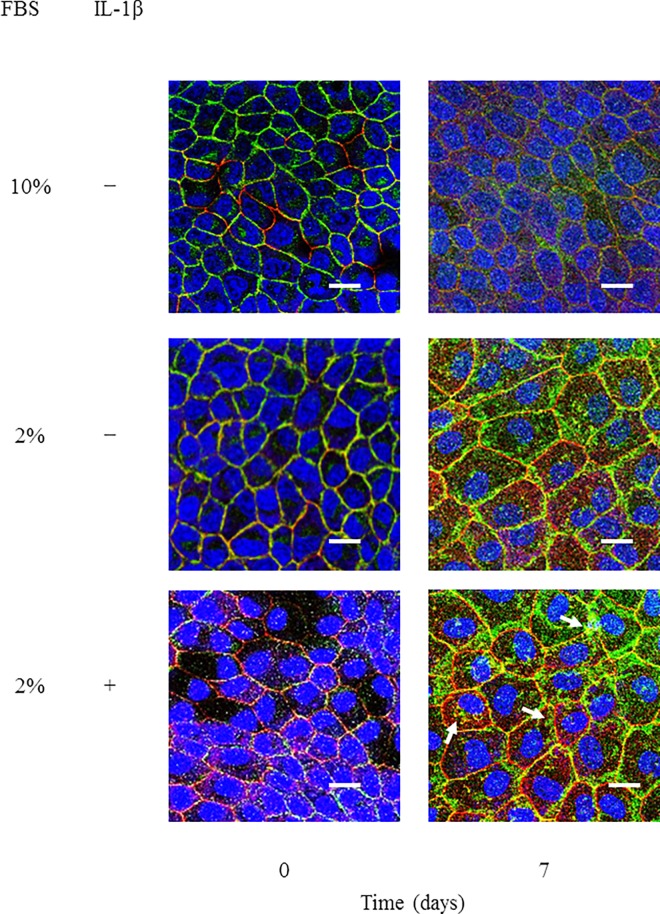
IL-1β induced a disruption in the localization of E-cadherin and ZO-1 and barrier function in MDCK cells. Cells were treated with culture medium containing 10% or 2% FBS in the presence or absence of 50 pM IL-1β for the indicated time periods. Then, the cells were labeled with fluorescent dye for nuclear staining (blue, nuclei) and antibodies against E-cadherin (green) and ZO-1 (red). The IL-1β-treated cells displayed a loss of membrane localization of E-cadherin and ZO-1. Spotty cytoplasmic localization patterns of E-cadherin and ZO-1 (arrows) were observed in the IL-1β-treated cells.

**Fig 2:**
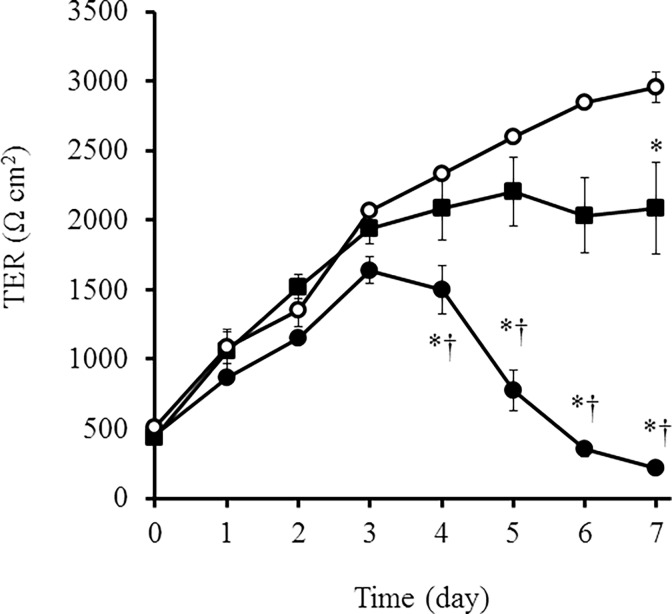
IL-1β induced a decrease in TER values. In cells treated with 10% FBS (open circles) and 2% FBS in the presence (closed circles) or absence (closed squares) of IL-1β (50 pM) for the indicated time periods, the barrier properties were monitored by measuring TER. Results are presented as mean ± SE from three independent experiments. *P < 0.05 vs. cells treated with 10% FBS (open circles). †*P* < 0.05 vs. cells treated with 2% FBS in the absence of IL-1β (closed squares).

Following an ischemic or toxic insult, inflammatory mediators are produced locally and contribute to kidney injury. In renal tubular cells, loss of cytoskeletal integrity and mislocalization of adhesion molecules have been found to be induced [22]. We examined the effect of IL-1β in the localization of proteins of adherence and tight junctions. E-cadherin and ZO-1 were localized at the membrane in control cells (Fig 1 and S1 Fig). However, in IL-1β-treated cells, loss of membrane localization and aberrant cytoplasmic localization of E-cadherin and ZO-1 were observed, whereas a lesser effect was seen in the cells cultured without IL-1β (Fig 1 and S1 Fig). Next, we examined the effect of IL-1β on the barrier properties of MDCK cells by monitoring TER, because the proper function of renal tubular cells is to act as a barrier to control paracellular transport of water, electrolytes, and macromolecules [23, 24]. As shown in Fig 2, the TER values increased in a time-dependent manner in control cells; however, the TER values in cells cultured with IL-1β decreased significantly after 5 days, although the values were slightly decreased in the cells cultured with medium containing 2% FBS.

### IL-1β induces NGAL mRNA expression and protein secretion in MDCK cells

**Fig 3:**
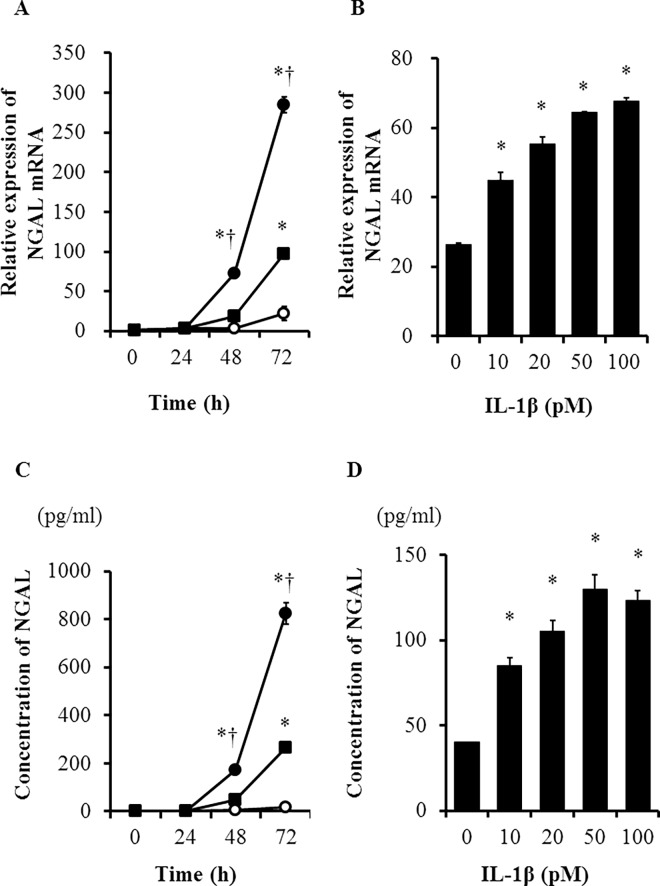
IL-1β induced NGAL mRNA expression and protein secretion in MDCK cells. (A) IL-1β induced NGAL mRNA expression in a time-dependent manner. The cells were treated with 10% FBS (open circles) and 2% FBS in the presence (closed circles) or absence (closed squares) of IL-1β (50 pM) for the indicated time periods. *P < 0.05 vs. cells treated with 10% FBS (open circles). †*P* < 0.05 vs. cells treated with 2% FBS in the absence of IL-1β (closed squares). (B) IL-1β treatment for 48 h induced an increase in NGAL mRNA expression dose dependently. *P < 0.05, compared with untreated cells. (C) IL-1β induced NGAL secretion time dependently. NGAL secretion was measured by ELISA in cells treated with 10% FBS (open circles) and 2% FBS in the presence (closed circles) or absence (closed squares) of IL-1β (50 pM) for the indicated time periods. *P < 0.05 vs. cells treated with 10% FBS (open circles). †*P* < 0.05 vs. cells treated with 2% FBS in the absence of IL-1β (closed squares). (D) IL-1β treatment for 48 h induced an increase in NGAL secretion dose dependently. *P < 0.05, compared with untreated cells.

NGAL has been reported to be expressed in epithelial cells under conditions of inflammation or malignancy [16, 19]. It is also involved in kidney development and homeostasis [25]. Therefore, we examined the effect of IL-1β on the expression of NGAL mRNA in MDCK cells by real-time RT-PCR. NGAL mRNA expression increased 48 h after treatment with IL-1β (Fig 3A) in a dose-dependent manner (Fig 3B). We also determined the concentration of NGAL protein in the cell culture medium. IL-1β induced an increase in the concentration of NGAL in the culture medium 48 h after treatment (Fig 3C) in a dose-dependent manner, similar to the increase in NGAL mRNA expression (Fig 3D). These observations indicate that NGAL expression and secretion are induced prior to the barrier dysfunction in cells treated with IL-1β, suggesting that NGAL could be a helpful biomarker for the early diagnosis of kidney injury.

### p38 and ERK1/2 are involved in IL-1β-induced NGAL expression in MDCK cells

**Fig 4:**
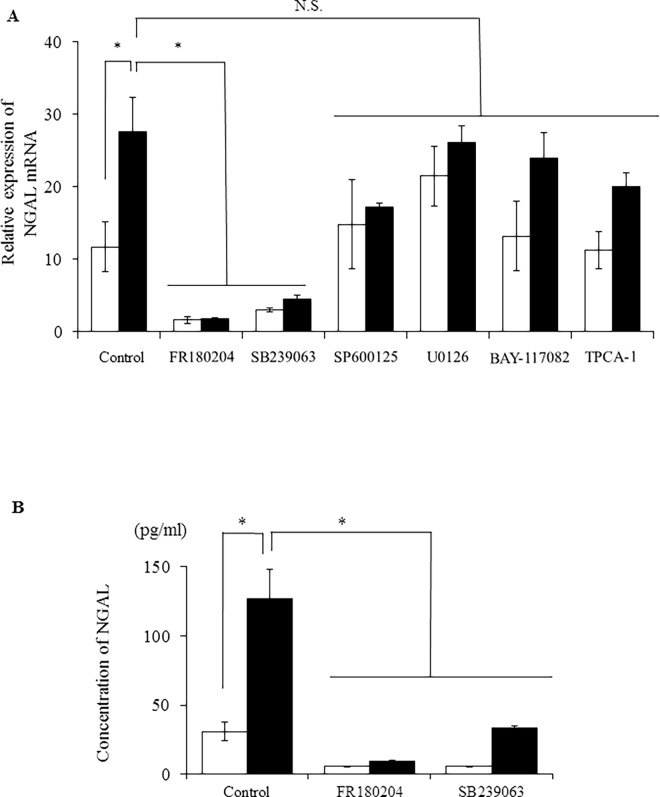
p38 and ERK1/2 inhibitors attenuated IL-1β-induced NGAL mRNA expression (A) and protein secretion (B) in MDCK cells. After pre-treatment with or without the ERK1/2 inhibitor FR180204 (25 μM), p38 inhibitor SB239063 (20 μM), JNK inhibitor SP600125 (10 μM), MEK inhibitor U0126 (20 μM), IKKα/β inhibitor BAY-117082 (10 μM), or IKKβ inhibitor TPCA-1 (10 μM) for 1 h, the cells were incubated with (black column) or without (white column) IL-1β (50 pM) for 48 h. Results are presented as mean ± SE from three independent experiments. *P < 0.05.

IL-1β has been demonstrated to activate MAPK and NF-κB signaling pathways in many types of cells, including human mesangial cells [18]. Thus, we examined whether MAPK or NF-κB signaling pathways were involved in the induction of NGAL expression by IL-1β in MDCK cells using pharmacological inhibitors of JNK, p38, and MEK/ERK1/2, the three main MAPK pathways [26, 27]. When the cells were treated with the ERK1/2 inhibitor FR180204 (25 μM) or the p38 MAPK inhibitor SB239063 (20 μM) for 1 h, IL-1β failed to enhance NGAL mRNA expression (Fig 4A). In contrast, SP600125 (10 μM) and U0126 (20 μM), inhibitors of JNK and MEK, respectively, had weaker effects on IL-1β-induced NGAL mRNA expression (Fig 4A). IκB kinases α/β (IKKα/β) are enzymes that activate the NF-κB signaling pathway. BAY-117082 (10 μM) or TPCA-1 (10 μM), inhibitors of IKKα/β or IKKβ, respectively, had a lesser effect on IL-1β-induced NGAL mRNA expression (Fig 4A). The expression of the housekeeping gene *TBP* was unaffected by the inhibitor. These inhibitors had no effect on the viability of MDCK cells. Next, we examined the effects of ERK1/2 and p38 inhibitors on IL-1β-induced NGAL protein secretion. Both FR180204 and SB239063 inhibited IL-1β-induced NGAL protein secretion (Fig 4B). These observations suggest that ERK1/2 and p38 are involved in IL-1β-induced mRNA expression and protein secretion of NGAL.

### NGAL preserves the barrier function in IL-1β-treated MDCK cells

**Fig 5:**
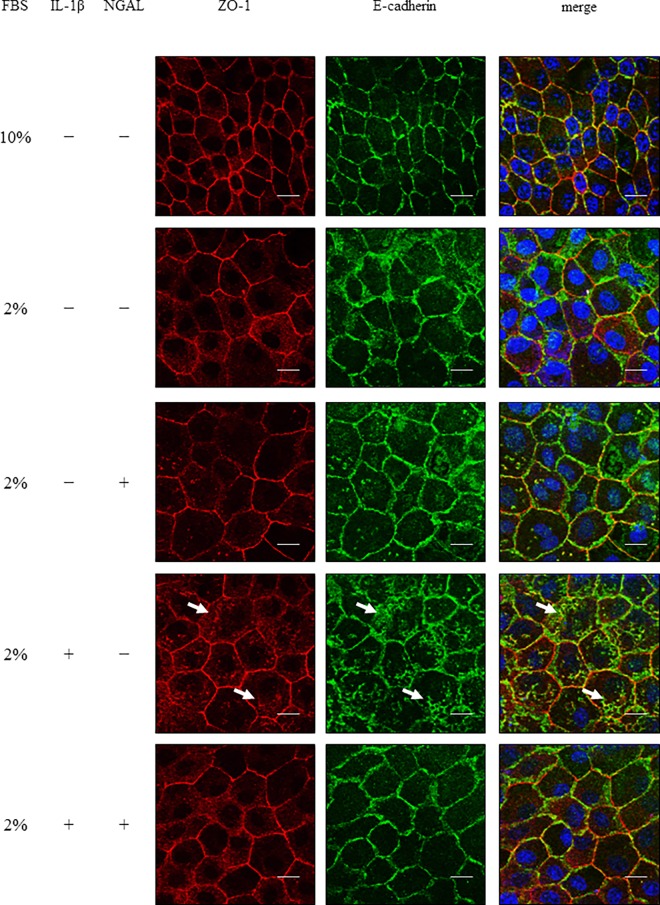
Recombinant NGAL prevented IL-1β-induced disruption in the localization of adherence and tight junction proteins in MDCK cells. After treatment with or without IL-1β (50 pM) in culture medium containing 10% or 2% FBS in the presence or absence of 50 ng/ml recombinant NGAL for 7 days, the cells were labeled with fluorescent dye for nuclear staining (blue, nuclei) and antibodies against E-cadherin (green) and ZO-1 (red). The IL-1β-treated cells displayed a loss of membrane localization of E-cadherin and ZO-1. Spotty cytoplasmic localization patterns of E-cadherin and ZO-1 (arrows) were observed in the IL-1β-treated cells. However, in the presence of recombinant NGAL, IL-1β-induced loss of membrane localization and spotty cytoplasmic localization of E-cadherin and ZO-1 were attenuated.

**Fig 6:**
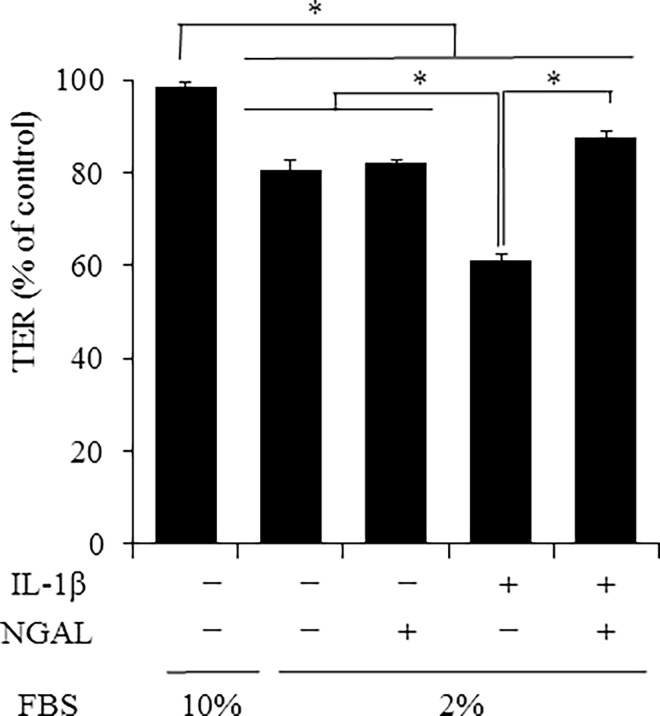
Recombinant NGAL prevented IL-1β-induced decrease in TER values. After treatment with or without IL-1β (50 pM) in culture medium containing 10% or 2% FBS in the presence or absence of 50 ng/ml recombinant NGAL for 7 days, the barrier function was monitored by measuring TER values. Results are presented as mean ± SE from three independent experiments. *P < 0.05.

Several studies have reported a relationship between NGAL and kidney diseases [16, 28]. However, besides the role of NGAL as a biomarker, its functions remain unclear [18]. We investigated the effect of NGAL on IL-1β-induced disturbance of E-cadherin and ZO-1 membrane localization. As Fig 5 summarizes, IL-1β induced a loss of membrane localization and cytoplasmic localization of E-cadherin and ZO-1. However, in the presence of recombinant NGAL, IL-1β failed to induce the loss of membrane localization and cytoplasmic localization of E-cadherin and ZO-1, similar to the control. Next, we examined the effect of recombinant NGAL on TER values. IL-1β treatment for 7 days resulted in a decrease in TER values, but it was prevented in the presence of recombinant NGAL (Fig 6). These observations strongly suggest that NGAL protects the function of renal tubular cells from injury by preserving the localization of adherence and tight junction proteins.

## Discussion

Epithelial-mesenchymal transition (EMT) is a biological process that induces the transition of epithelial cells into mesenchymal fibroblast-like cells. EMT is characterized by the downregulation of the key epithelial protein E-cadherin [29–31]. The downregulation of cell-surface E-cadherin is observed in kidney diseases, and EMT is considered to play an important role in the progression and development of AKI [32–34]. In various epithelial cells such as corneal epithelial cells, mesothelial cells, retinal endothelial cells, and oral squamous carcinoma cells, IL-1β has been reported to disrupt the intercellular junctions and exert profibrotic activity [35–38]. In a rat kidney epithelial cell line (NRK52E cells), IL-1β was found to decrease the expression of E-cadherin [29]. In this study, we demonstrated that IL-1β induced a disturbance in the localization of E-cadherin and ZO-1 and a disruption in the barrier function of MDCK cells. These observations suggest that IL-1β contributes to the development of AKI by mediating inflammation and EMT.

Serum creatinine and BUN have routinely been used to diagnose AKI. However, serum creatinine levels are affected by non-renal factors such as age, sex, muscle mass, nutritional status, infection, volume of distribution, and medications [15]. BUN levels are also influenced by non-renal factors such as protein intake, catabolic state, upper gastrointestinal bleeding, volume status, and therapy with high-dose steroids [15]. Therefore, highly sensitive, specific, and clinically available biomarkers for AKI have been investigated [16]. NGAL expression has been detected in the kidney, and NGAL locally produced in the distal tubule and collecting ducts is excreted into urine [16, 17]. In mouse models of ischemic- or cisplatin-induced renal injury, NGAL has been detected in urine [39, 40]. The upregulation of urinary NGAL associated with AKI after cardiac surgery has been reported [28]. The upregulation of urinary NGAL has also been reported to correlate with the severity of AKI. In a study on children with diarrhea-associated hemolytic uremic syndrome, urinary NGAL was reported to predict the severity of AKI and dialysis requirement [41]. In AKI patients, after cardiopulmonary bypass surgery, urinary NGAL levels have been found to correlate with the severity and duration of AKI, length of hospital stay, dialysis requirement, and death [42]. In this study, we observed that IL-1β-induced NGAL mRNA expression and secretion occurred prior to morphological and functional changes of MDCK cells. Therefore, it is conceivable that NGAL is an early biomarker for diagnosing AKI.

IL-1β-induced NGAL expression via the activation of NF-κB and MAPK signaling pathways has been demonstrated. In A549 cells, the induction of the NF-κB cofactor IκB-ζ is necessary for IL-1β-induced NGAL expression [43]. In human mesangial cells, IL-1β-induced ERK activation contributes to IL-1β-induced NGAL expression [18]. We demonstrated that ERK and p38 MAPK, but not NF-κB, contribute to IL-1β-induced NGAL expression in MDCK cells. Therefore, the signaling pathways activated in this process appear to depend on the type of cell and species.

In human lung cancer cells, the overexpression of NGAL significantly reduces cytotoxic drug-induced cell death [20]. In Ras-transformed mouse breast cancer cells, NGAL was found to diminish invasiveness and metastasis [44]. In rat renal tubular cells, NGAL promotes cell proliferation and reduced apoptosis [22]. In addition, NGAL was observed to protect the kidney and ease azotemia after ischemia-reperfusion-induced AKI [45]. These observations suggest that NGAL plays a protective role against AKI. We showed that exogenous NGAL significantly attenuated the disruption in the localization of junction proteins and the barrier function induced by IL-1β in MDCK cells. Our observations support the hypothesis that NGAL functions to protect against kidney injury.

It is still obscure how exogenous NGAL treatment protects the function of junctions in MDCK cells. In AKI model mice, NGAL induced renal cell regeneration via the downregulation of inflammatory cytokines [46]. In Ras-transformed cells, NGAL antagonized the Ras/Raf/MEK/ERK signaling pathway [44]. Because IL-1β has been reported to induce the expression of the inflammatory marker COX-2 via ERK activation in canine dermal fibroblasts [47], we examined the effect of exogenous NGAL on IL-1β-induced mRNA expression of inflammatory markers such as COX-2 and IL-8 in MDCK cells. However, NGAL had no effect on IL-1β-induced expression of COX-2 and IL-8 mRNAs (data not shown). Therefore, it is unlikely that the inhibition of the function of inflammatory cytokines or MAPK activation causes the protective effect of NGAL. On the other hand, the activation of the NGAL receptor SLC22A17 resulted in a reduction in apoptosis through the induction of the expression of the anti-apoptotic factor Bcl-2 and reduction of the expression of the pro-apoptotic factors Bax, Fas, and FasL in rat renal tubular cells [48]. The activation of SLC22A17 was suggested to induce the expression of antioxidant enzymes such as heme oxygenase-1 (HO-1) to protect cells from reactive oxygen species [49]. In AKI model mice, exogenous administration of NGAL ameliorated the structural and functional damage to kidneys caused by ischemic injury [50, 51], and HO-1 stimulated re-epithelialization in the tubular lamina [52]. Therefore, it is conceivable that the expression of antioxidant enzymes by the activation of NGAL receptors is involved in NGAL function as a protective factor against oxidative stress. Although we observed the expression of the NGAL receptor SLC22A17 in MDCK cells (data not shown), further studies regarding the relationship between NGAL and expression of antioxidant enzymes are underway in our laboratory.

## Conclusions

We demonstrated that IL-1β induces the upregulation of NGAL via p38 and ERK signaling pathways in MDCK cells, and that exogenous NGAL protects the function of intercellular junctions in IL-1β-treated MDCK cells. Our observations provide new insights into the role of NGAL as an early biomarker and as a target for the treatment of AKI.

## Supporting Information

S1 Fig Time-dependent changes of E-cadherin and ZO-1 localization in IL-1β-treated MDCK cells. Cells were treated with the medium containing 10% or 2% FBS in the presence or absence of 50 pM IL-1β for indicated time periods, and were labeled with the fluorescent dye for nuclear staining (blue, nuclei), and antibodies against E-cadherin (green) and ZO-1 (red).
(TIF)
