# Supplementary figures and images for: Expression and Function of Interleukin-1β-Induced Neutrophil Gelatinase-Associated Lipocalin in Renal Tubular Cells
Source: PLoS One. 2016 Nov 16;11(11):e0166707. doi: 10.1371/journal.pone.0166707 (PMC5112913; doi:10.1371/journal.pone.0166707)

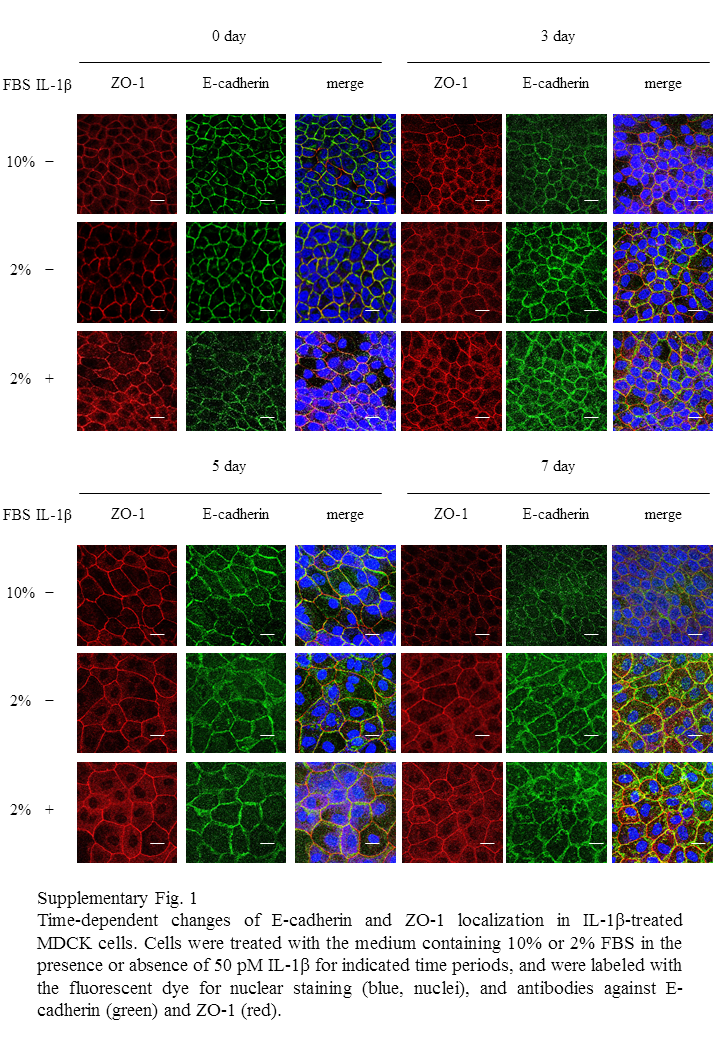

Supplement: S1 Fig — Cells were treated with the medium containing 10% or 2% FBS in the presence or absence of 50 pM IL-1β for indicated time periods, and were labeled with the fluorescent dye for nuclear staining (blue, nuclei), and antibodies against E-cadherin (green) and ZO-1 (red). (TIF) [file pone.0166707.s001.tif]
